# Mesonephric Adenocarcinoma of the Uterine Fundus Exhibiting High ^18^F-FDG Uptake

**DOI:** 10.3390/diagnostics10090729

**Published:** 2020-09-21

**Authors:** Hyung Kyung Kim, Kyu Yeoun Won, Chanwoo Kim

**Affiliations:** 1 Department of Pathology, Kyung Hee University Hospital at Gangdong, Kyung Hee University School of Medicine, Seoul 05278, Korea; hyungkyungjkim@gmail.com; 2 Department of Nuclear Medicine, Kyung Hee University Hospital at Gangdong, Kyung Hee University School of Medicine, Seoul 05278, Korea

**Keywords:** mesonephric adenocarcinoma, uterine fundus, ^18^F-FDG PET/CT

## Abstract

Mesonephric adenocarcinoma is a rare tumor that is considered to develop from mesonephric remnants of the female genital tract. This tumor usually occurs in the lateral wall of the uterine cervix. Herein, we present an exceptionally rare case of mesonephric adenocarcinoma located in the uterine fundus. The tumor exhibited intense hypermetabolism on ^18^F-FDG PET/CT. Based on the characteristic histologic features and immunohistochemical phenotypes, the diagnosis of mesonephric adenocarcinoma was confirmed. The patient underwent hysterectomy with bilateral salpingo-oophorectomy and pelvic lymph node dissection, and no lymph node or distant metastasis was identified. After 20 months of surveillance without adjuvant therapy, she remains free of relapse.

**Figure 1:**
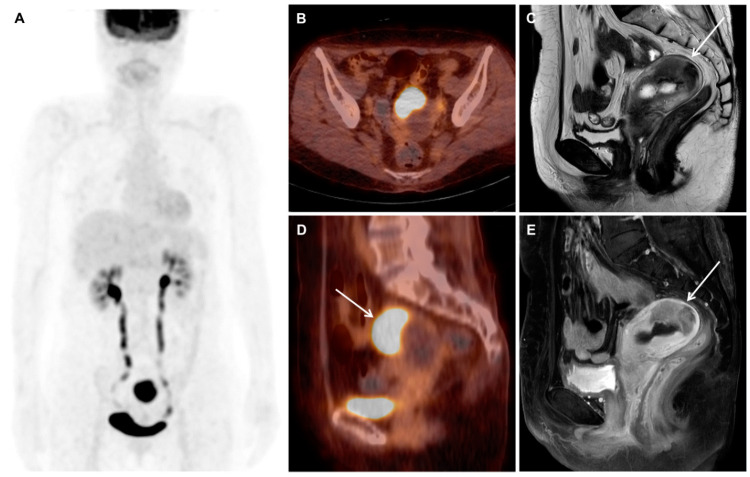
A 67-year-old postmenopausal woman who presented with vaginal bleeding underwent hysteroscopy, which revealed a protruding mass in the uterine cavity. Hysteroscopic resection was done and pathologic examination suggested a diagnosis of endometrioid adenocarcinoma with FIGO grade I. Carbohydrate antigen 125 (CA 125) was within the normal range (15.5 U/mL). She underwent an ^18^F-FDG PET/CT scan and a pelvic MRI scan for tumor staging. The ^18^F-FDG PET/CT images (**A**, **B**, **D**; maximum intensity projection image, transaxial and sagittal fused PET/CT images) showed an intense hypermetabolic mass (arrow, SUVmax of 23.1) in the uterine fundus and no evidence of metastasis. The MRI images (**C**, **E**; T2-weighted image and gadolinium-enhanced T1-weighted image) showed a mass in the uterine fundus, which was confined to the myometrium (arrows). Since the epicenter of the tumor was in the myometrium, leiomyosarcoma rather than endometrial cancer was suspected. As there was no evidence of distant metastasis, she underwent hysterectomy with bilateral salpingo-oophorectomy and pelvic lymph node dissection.

**Figure 2:**
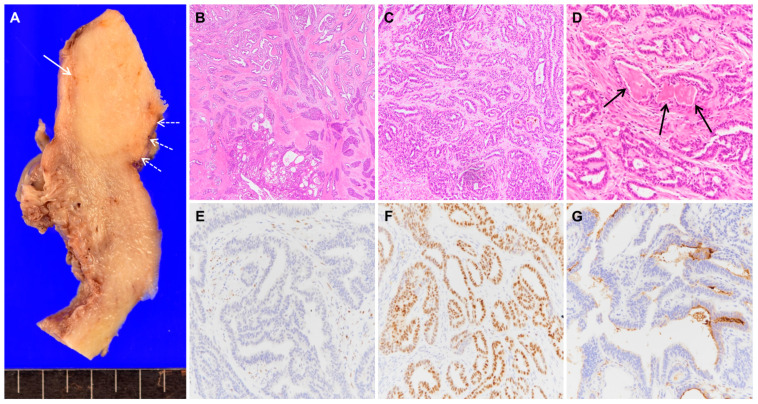
The macroscopic image of the hysterectomy specimen showed the cut surface of the ovoid yellowish solid mass (**A**, solid arrow) in the myometrium, which was not in continuity with the atrophic endometrium (**A**, dashed arrows). The microscopic examination revealed that the tumor comprised various architectural structures that showed tubular, papillary, solid, or retiform patterns (**B**, magnification ×20). The most common growth pattern (**C**, magnification ×100) was small tubular glands lined by cuboidal epithelium containing eosinophilic secretion (**D**, arrows, magnification ×200). By immunohistochemical studies, tumor cells were negative for ER (**E**, magnification ×200) and progesterone receptor (PR), and positive for GATA3 (**F**, magnification ×200) and PAX2. The tumor showed luminal expression of CD10 (**G**, magnification ×200) in the tubular structures but was negative for calretinin. Based on these characteristic morphologic features and immunohistochemical profiles [1,2], a diagnosis of mesonephric adenocarcinoma was made. The tumor did not invade the cervix or uterine serosa. There were no definite foci of mesonephric remnants or hyperplasia around the tumor. No lymph node metastasis was identified in the pelvis. Mesonephric adenocarcinoma is a rare tumor, which is assumed to arise from mesonephric (Wolffian) remnants of the female genital tract. The remnants are usually located in the lateral wall of the cervix in which the majority of mesonephric adenocarcinomas occur but may also be found within the uterine corpus, vagina, and ovary [3,4]. Mesonephric adenocarcinoma of the uterine corpus is thus even rarer with only few cases reported in the literature [1,4]. They were typically found in the posterior or lateral myometrium of the uterine body [1,4], where mesonephric remnants are theoretically present. However, this case demonstrates the mesonephric adenocarcinoma located in the uterine fundus, which has never been reported to the best of our knowledge. As in this case, the diagnosis of mesonephric adenocarcinoma on the basis of endometrial biopsy is challenging. Morphologically, mesonephric adenocarcinoma has characteristic structural heterogeneity with an admixture of tubular, glandular, papillary, retiform, and solid growth patterns [1,3]. Accordingly, the biopsy specimens, which contain only a small part of tumors, are less likely to include all the diverse architectures. Mesonephric adenocarcinoma has a characteristic immunophenotype as well. Although CD10 and calretinin have previously been used to support the mesonephric differentiation, their clinical utility is hampered by relatively low sensitivity and specificity [2,4]. In the present case, the tumor was positive for CD10 but negative for calretinin. Recently, GATA3 was reported to display the best performance among other markers for identifying mesonephric adenocarcinoma [2]. The pathogenesis of mesonephric adenocarcinoma of the uterine corpus is still in debate [4,5,6]. The tumor is believed to develop from the remnants of the Wolffian duct, which are predisposed to be located in the lateral myometrium. On the other hand, the endometrial origin of the tumor has also been proposed [5,6], hence it can be considered a Müllerian adenocarcinoma with subsequent mesonephric differentiation and myometrial invasion. Recently, the latter has been termed mesonephric-like adenocarcinoma [6]. In this case, the tumor seems to be originated from mesonephric remnants, because it is entirely confined to the myometrial layer, the diagnosis, therefore, should be mesonephric adenocarcinoma. Nonetheless, there is still a possibility that the tumor arose from adenomyosis, which indicates the endometrial origin. However, adenomyosis was not found in the myometrium. Mesonephric-like adenocarcinoma of the endometrium showed an aggressive clinical behavior with significantly shorter survival than uterine serous carcinoma, an aggressive form of endometrial cancer [7]. The metastatic rate of mesonephric adenocarcinoma of the uterine corpus was also higher, especially to the lungs, compared with mesonephric adenocarcinoma of the cervix [1]. Despite the poor prognosis reported, at follow-up 20 months after the surgery, the patient of this case remains free of disease.

## References

[1] Na K., Kim H.S. (2019). Clinicopathologic and molecular characteristics of mesonephric adenocarcinoma arising from the uterine body. Am. J. Surg. Pathol..

[2] Pors J., Cheng A., Leo J.M., Kinloch M.A., Gilks B., Hoang L. (2018). A comparison of GATA3, TTF1, CD10, and calretinin in identifying mesonephric and mesonephric-like carcinomas of the gynecologic tract. Am. J. Surg. Pathol..

[3] Howitt B.E., Nucci M.R. (2018). Mesonephric proliferations of the female genital tract. Pathology.

[4] Ando H., Watanabe Y., Ogawa M., Tamura H., Deguchi T., Ikeda K., Fujitani M., Shioji M., Tsujie T., Doi R. (2017). Mesonephric adenocarcinoma of the uterine corpus with intracystic growth completely confined to the myometrium: A case report and literature review. Diagn. Pathol..

[5] Yano M., Shintani D., Katoh T., Hamada M., Ito K., Kozawa E., Hasegawa K., Yasuda M. (2019). Coexistence of endometrial mesonephric-like adenocarcinoma and endometrioid carcinoma suggests a Müllerian duct lineage: A case report. Diagn. Pathol..

[6] McFarland M., Quick C.M., McCluggage W.G. (2016). Hormone receptor-negative, thyroid transcription factor 1-positive uterine and ovarian adenocarcinomas: Report of a series of mesonephric-like adenocarcinomas. Histopathology.

[7] Euscher E.D., Bassett R., Duose D.Y., Lan C., Wistuba I., Ramondetta L., Ramalingam P., Malpica A. (2020). Mesonephric-like carcinoma of the endometrium: A subset of endometrial carcinoma with an aggressive behavior. Am. J. Surg. Pathol..

